# Microparticles from Patients with Metabolic Syndrome Induce Vascular Hypo-Reactivity via Fas/Fas-Ligand Pathway in Mice

**DOI:** 10.1371/journal.pone.0027809

**Published:** 2011-11-15

**Authors:** Abdelali Agouni, Pierre-Henri Ducluzeau, Tarek Benameur, Sébastien Faure, Martina Sladkova, Lucie Duluc, Georges Leftheriotis, Olga Pechanova, Mirela Delibegovic, Maria Carmen Martinez, Ramaroson Andriantsitohaina

**Affiliations:** 1 INSERM, U694, Angers, France; Université d'Angers, Angers, France; 2 Institute of Biological and Environmental Sciences, University of Aberdeen, Aberdeen, Scotland, United Kingdom; 3 Département d'Endocrinologie et Diabétologie, CHU d'Angers, Angers, France; 4 Institute of Normal and Pathological Physiology, Slovak Academy of Sciences, Bratislava, Slovak Republic; 5 INSERM, U771, Angers, France; Leiden University Medical Center, The Netherlands

## Abstract

Microparticles are membrane vesicles with pro-inflammatory properties. Circulating levels of microparticles have previously been found to be elevated in patients with metabolic syndrome (MetS). The present study aimed to evaluate the effects of *in vivo* treatment with microparticles, from patients with MetS and from healthy subjects (HS), on *ex vivo* vascular function in mice. Microparticles isolated from MetS patients or HS, or a vehicle were intravenously injected into mice, following which vascular reactivity in response to vasoconstrictor agonists was assessed by myography with respect to cyclo-oxygenase pathway, oxidative and nitrosative stress. Injection of microparticles from MetS patients into mice induced vascular hypo-reactivity in response to serotonin. Hypo-reactivity was associated with up-regulation of inducible NO-synthase and increased production of NO, and was reversed by the NO-synthase inhibitor (N^G^-nitro-L-arginine). The selective COX-2 inhibitor (NS398) reduced the contractile effect of serotonin in aortas from mice treated with vehicle or HS microparticles; however, this was not observed within mice treated with MetS microparticles, probably due to the ability of MetS microparticles to enhance prostacyclin. MetS microparticle-mediated vascular dysfunction was associated with increased reactive oxygen species (ROS) and enhanced expression of the NADPH oxidase subunits. Neutralization of the pro-inflammatory pathway Fas/FasL completely prevented vascular hypo-reactivity and the ability of MetS microparticles to enhance both inducible NO-synthase and monocyte chemoattractant protein-1 (MCP-1). Our data provide evidence that microparticles from MetS patients induce *ex vivo* vascular dysfunction by increasing both ROS and NO release and by altering cyclo-oxygenase metabolites and MCP-1 through the Fas/FasL pathway.

## Introduction

Metabolic syndrome (MetS) is associated with glucose intolerance, obesity, ageing, elevated blood pressure and dyslipidaemia, all of which are risk factors associated with cardiovascular morbidity and mortality [1], [2]. The prevalence of MetS is increasing and continues to provide challenges for medical research beyond its clinical and public health importance.

The pathophysiology of MetS seems to be largely attributable to insulin resistance with the implication of excessive flux of fatty acids [1], [2], but also to a pro-inflammatory state resulting from the production of cytokines from adipocytes and macrophages [1], [3], [4], [5]. Thus, increased inflammatory factors and reactive oxygen species (ROS) are associated with detrimental cardiovascular alterations linked to MetS. Inflammation is orchestrated by the interactions between inflammatory cells (such as leukocytes) and vascular cells (endothelial and smooth muscle cells) which under activation or apoptosis (for example) lead to the release of circulating microparticles (MPs) [6], [7].

MPs are membrane vesicles with pro-coagulant and pro-inflammatory properties [6], [7]. The mechanism of MP formation is complex and has yet to be clearly elucidated, due to cell type and stimuli specificity. However, evidence suggest that following cell activation or apoptosis, MP formation occurs due to the sustained elevation in cytosolic calcium concentration in addition to the consequent activation of calpain and protein kinases and the inhibition of phosphatases. In addition to MP formation, these changes result in cytoskeletal reorganization and membrane blebbing [6], [8], [9], [10]. The mechanism of MPs clearance from the circulation is currently unknown, but due to their small size, MPs are believed to be more readily diffusible than cells, and are able to escape phagocytosis [11].

MPs are present in the blood of healthy individuals, however MP formation increases with diseased states, such as MetS [12], sepsis [13], preeclampsia [14], and sleep apnea syndrome [15]. MP release also increases in various clinical situations associated with thrombosis, as well as in diabetic patients, thereby strengthening the hypothesis that MPs may play an important role in these diseases. Indeed, MPs can be considered as vectors of biological messages, such as induction of endothelial and vascular dysfunctions or platelet activation [6], [7].

Recently, we reported that MetS patients display elevated circulating levels of MPs compared to healthy subjects, especially those from pro-coagulant (Annexin V^+^), endothelial, platelet and erythrocyte origins; furthermore these MPs are associated with endothelial dysfunction both *in vitro* and *in vivo* [12]. Moreover, Arteaga et al. [16] reported a marked elevation of endothelial-derived MP levels as well as activation markers of both leukocytes and platelets in patients with MetS compared to controls. In addition, they observed an increase in binding of endothelial- and platelet-derived MPs to leukocytes within MetS patients. Finally, Chironi et al. [17] found that leukocyte-derived MP level was higher in MetS patients than in healthy individuals and that leukocyte-derived MP level increased concomitantly with the number of MetS components.

Nevertheless, the effects of MPs from MetS patients on vascular reactivity in response to vasoconstrictor agonists have not yet been assessed. Therefore, the aims of the present study were to: 1) assess the effect of circulating MPs from MetS patients on vascular reactivity with special interest in oxidative and nitrosative stresses *in vivo*, and 2) identify cellular mechanism(s) underlying these effects. Amongst the known pro-inflammatory pathways, we focused our interest on the potential involvement of Fas/FasL pathway. Fas is a type I membrane protein belonging to the TNF receptor family that initiates an apoptotic signal when bound to its ligand, FasL [18]. Functionally, FasL has been demonstrated to be a pro-inflammatory factor that is implicated in pathophysiological processes of various cardiovascular diseases such as coronary heart disease, arteriosclerosis, as well as ischemia-reperfusion injury [19]. Fas/FasL interaction is involved in apoptosis of vascular smooth muscle cells [20] and the expression of a specific program of inflammatory genes in the same cells [21], [22]. It should be noted that we previously reported that lymphocytic MPs interact with smooth muscle cells through the Fas/FasL pathway, evoke NF-κB activation, which in turn up-regulates iNOS and COX-2 expression leading to the production and release of the vasodilatory factors, NO and prostacyclin [23].

## Methods

### Ethics Statement

The human study was approved by the Ethics Committee of the University Hospital of Angers (France) as the NUMEVOX cohort (registration number: 200704256). The NUMEVOX cohort is also referenced at Clinicaltrials.gov (Impact of Adipose Tissue Repartition on the Time Course of Vascular Dysfunction - the NUMEVOX Cohort). The investigation conforms to the principles outlined in the Declaration of Helsinki. All subjects agreed to participate in the follow-up study by written consent.

The University of Angers Ethics Committee approved the animal protocol followed in the present study (CEEA.2009.9). All animal studies were carried out using approved institutional protocols and conformed by the *Guide for the Care and Use of Laboratory animals* published by US National Institutes of Health (NIH Publication No. 85-23, revised 1996).

### Subjects

We included patients with MetS from the Department of Endocrinology and Nutrition of the University Hospital of Angers. Patients were eligible for inclusion, according to the National Cholesterol Education Program-Adult Treatment Panel III (NCEP-ATP III) [24], [25], when they had at least three criteria out of the five following: (I) waist circumference >102 or 88 cm for men and women, respectively; (II) high systolic and diastolic pressures >130/85 mm Hg; (III) fasting glycemia >5.5 mmoll^−1^; (IV) triglycerides >1.65 mmoll^−1^ and (V) high-density lipoprotein (HDL) <1 mmoll^−1^ in men or <1.3 mmoll^−1^ in women. Patients with a history of cardiovascular diseases, pre-existent chronic inflammatory disease and/or cancer were not included. Normal controls consisted of subjects who met less than two of the MetS criteria. These subjects are referred here as healthy subjects (HS).

### MP isolation

Peripheral blood (20 ml) from HS and MetS patients was collected in EDTA-treated tubes (Vacutainers, Becton Dickinson, Le Pont de Claix, France) from a peripheral vein using a 21-gauge needle to minimize platelet activation and were processed for assay within 2 h [12], [13]. After a 20 min centrifugation (270×*g*), platelet-rich plasma was separated from whole blood. Platelet-rich plasma was then centrifuged for 20 min (1500×*g*) to obtain platelet-free plasma (PFP). Two hundred µl of PFP was frozen and stored at –80°C. Remaining PFP (MP-containing) was spun down at 21,000×*g* for 45 min to pellet MPs. The MP pellet was then centrifuged twice at 14,000×*g* for 45 min. Finally, the MP pellet was resuspended in 200 µl of 0.9% saline salt solution and stored at 4°C, as previously described [12], [13]. For each healthy subject or MetS patient, MP concentration in plasma was determined by flow cytometry. Briefly, an equal volume of sample and Flowcount beads (Beckman Coulter, Villepinte, France) were added and samples were analyzed in a flow cytometer 500 MPL System (Beckman Coulter) [12], [13]. Levels of endotoxin were assessed in all MP preparations with the Limulus amebocyte lysate kit QCL- 1000 (Lonza, Verviers, Belgium) and were found to be below the lower detection limit of the kit (<0.1 endotoxin Uml^−1^).

For animal treatments, we injected the mice with either supernatants corresponding to the last MP washing medium (vehicle) or MPs at the circulating level detected in each donor (HS and MetS patients). The values of circulating MPs detected in donors and injected into mice ranged from 1264 and 16734 MPsµl^−1^ of plasma for HS and 2575 and 23266 MPsµl^−1^ of plasma for MetS patients. Characterization of MPs by flow cytometer is shown in Table S1. As previously shown [12], total circulating levels of MPs and platelet-, endothelial- and red cell-derived MPs were significantly enhanced when compared with HS MPs.

### Vascular reactivity

Male Swiss mice (8–10 weeks old) were treated *in vivo* by intravenous (*i.v.*) injection into the tail vein with either vehicle or MPs at the circulating levels of MPs detected in the blood of each patient (MetS MPs) or healthy subject (HS MPs). Each mouse has been injected with MPs from one donor. Animals had continuous access to food and water throughout the experiment and were maintained on a 12:12 light∶dark cycle.

Due to the findings from our preliminary studies in previous projects investigating MP effects on vascular function that the 24 h duration was the best time to observe changes at the functional level for vascular function [23], [26], [27], we injected the mice with MPs or vehicle control 24 h prior to being sacrificed by CO_2_. After mice were culled, aortas were removed and cleaned of adhering fat and connective tissue and then cut into rings (1.5–2 mm long) that were mounted on a wire myograph filled with physiological salt solution as previously described [12], [13]. Concentration-response curves were constructed by cumulative application of serotonin (5-HT, 1 nmoll^−1^ to 10 µmoll^−1^; Sigma-Aldrich, St. Quentin, Fallavier, France) to vessels with functional endothelium in the absence or presence of the given inhibitor pre-incubated for 30 min: the NO-synthase inhibitor N^G^-nitro-L-arginine (L-NA, 100 µmoll^−1^; Sigma-Aldrich), the selective cyclooxygenase (COX)-2 inhibitor N-(2-cyclohexyloxy-4-nitrophenyl) methanesulfonamide (NS-398, 10 µmoll^−1^; Sigma-Aldrich), indomethacin, the non selective COX inhibitor (100 µmoll^−1^; Sigma-Aldrich). All inhibitors were used at maximal active concentrations at which they inhibit the release of either NO from all isoforms of NO-synthases, metabolites from COX-2 isoform or metabolites from COX in blood vessels, as reported in many of our previous studies [13], [23]. Higher concentrations of L-NA, NS-398 or indomethacin did not induce further inhibition.

The response to KCl (80 mmoll^−1^) containing thromboxane A_2_ agonist (9,11-dideoxy-11a,9a-epoxymethanoprostaglandin F2α) (U46619, 100 nmoll^−1^, Sigma-Aldrich) was also assessed in mice aortas in order to test their maximal contractile capacity.

In another set of experiments, MetS MPs were pre-incubated with human anti-FasL antibody (5 µg, BD Biosciences, San Jose, CA) for 1 h at 4°C to allow neutralization of MP FasL. After two washes with PBS in order to remove unbound anti-FasL antibody, MetS MPs were then injected i.v. into mice and 24 h later, vascular reactivity in response to 5-HT was assessed as described above.

### Determination of prostanoid production

Mice, both pre-treated with MPs and controls were culled and aortas were dissected, placed in phosphate saline solution PSS and treated with 5-HT (1 µmoll^−1^, 37°C, 30 min). After collection of PSS, prostacyclin was measured by enzyme immunoassays kits (Cayman Chemicals, Ann Arbor, MI). The concentration of prostanoids was expressed as pgml^−1^ mg^−1^ of tissue (dry weight).

### NO determination by electron paramagnetic resonance (EPR)

Animals were culled 24 h after administration of MPs or vehicle. For NO production, aortas were dissected and incubated for 30 min in Krebs-Hepes buffer containing: BSA (20.5 gl^−1^), CaCl_2_ (3 mmoll^−1^) and L-Arginine (0.8 mmoll^−1^). NaDETC (1.5 mmoll^−1^) and FeSO_4_.7H_2_O (1.5 mmoll^−1^) were separately dissolved under argon gas bubbling in 10 ml volumes of ice-cold Krebs-Hepes buffer. These were rapidly mixed to obtain Fe(DETC)_2_ solution (0.4 mmoll^−1^), which was added to the aortas and incubated for 45 min at 37°C. Aortas were immediately frozen in plastic tubes using liquid N_2_. NO measurement was performed on a table-top x-band spectrometer Miniscope (Magnettech, MS200, Berlin, Germany). Recordings were made at 77°K. Instrument settings were 10 mW of microwave power, 1 mT of amplitude modulation, 100 kHz of modulation frequency, 150 s of sweep time and 5 scans. Signals were quantified by measuring the total amplitude, after correction of baseline as done previously [13], [23], [28].

### Dihydroethidine (DHE) staining and reactive oxygen species (ROS) production

*In situ* production of ROS in the vessel wall was evaluated with the oxidative fluorescent dye DHE as previously described [29]. Briefly, aorta sections (7 µm) were mounted on glass slides and incubated with DHE (3 µmoll^−1^, 30 min, 37°C). A Solamere confocal equipment with a DLS-300 laser mounted on a Nikon Eclipse TE 2000-S inverted microscope was used for the optical sectioning of the tissue. Digital image recording was performed using the QED *in vivo* Software. Pixel quantification was executed by scanning densitometry (Image J software).

### Western Blotting

Each aorta was dissected, homogenized and lysed. Proteins (80 µg) were separated on 10% SDS-PAGE. Blots were probed with anti-inducible NOS (iNOS) (BD Biosciences), COX-1 and COX-2 (Santa Cruz Biotehnology, Santa Cruz, CA), Mn-superoxide dismutase (SOD), Cu/Zn-SOD, extracellular-SOD (Ec-SOD) (Stressgen Biotechnologies Corporation, Victoria, Canada), gp91^phox^, p47^phox^, p67^phox^ (BD Biosciences), Monocyte Chemotactic Protein (MCP)-1 (R&D systems, Abingdon, UK) antibodies. In another set of experiments, total proteins from MetS MPs were probed with anti-FasL antibody (BD Biosciences).

A polyclonal rabbit anti-human β-actin antibody (Sigma-Aldrich) was used as a loading control. Membranes were washed at least three times in Tris-buffer solution containing 0.05% Tween and incubated for 1 h at room temperature with the appropriate horseradish peroxidase (HRP)-conjugated secondary antibody (Amersham, Piscataway, NJ). Protein bands were detected by enhanced chemiluminescence plus (Amersham) according to the protocol of the manufacturer. Immunoblots were quantified by densitometric analysis (LAS3000 software, Fujifilm, Bois d'Arcy, France).

### Quantitative real time reverse transcription-polymerase chain reaction (RT-PCR) analysis

Aortas taken from mice injected either with vehicle, MetS MPs or HS MPs were cleaned, frozen in liquid N_2_ and used to investigate the expression of mRNA for IL-6, IL-8, IL-1α, IL-1β, IL-10, TNF-α, MCP-1, TGF-β1, TGF-β2 and TGF-β3 by RT-PCR. RT-PCR analyses were carried out using a Chromo 4^tm^ (Bio-Rad, Marnes-la-Coquette, France) and SYBR Green detection. Quantifications were realized according to the ΔCt method as previously described [30].

### Data analysis

Data are expressed as mean ± SEM, and *n* represents the number of mice. pD_2_ = −log EC_50_, EC_50_ being the molar concentration of the agonist that produces 50% of the maximal effect; EC_50_ values were calculated by log-log regression. Statistical analyses were performed using ANOVA, and Mann-Whitney U tests or ANOVA for repeated measures and subsequent Bonferroni post hoc test. *P*<0.05 was considered to be statistically significant.

## Results

As shown in Table 1, there were no significant differences between MetS patients and HS with respect to age. As expected, MetS patients showed greater BMI, visceral obesity (as measured by waist circumference), enhanced triglyceridemia, and increased blood pressure. HbA1c values were not higher than 7.5% both in MetS patients and HS. Insulin levels were significantly higher in MetS patients, indicating insulin resistance. In addition, MetS patients exhibited lower levels of adiponectin compared to control subjects, although leptin levels were similar in both groups, supporting insulin resistance within the MetS patients [31]. Over half of the MetS patients exhibited 4 out of 5 MetS criteria (Table 1).

**Table 1:** Baseline characteristics of subjects.

	Control subjects	MetS patients
**Number**	17	21
**Mean age (years)**	54±2	56±2
**Sex ratio (male/female)**	12/5	17/4
**BMI (kg/m^2^)**	28±1.3	34±1.1^c^
**Waist circumference (cm)**	94±3.2	113±2.3^c^
**Ratio Waist/Hips**	0.95±0.02	1.01±0.01^a^
**Systolic blood pressure (mm Hg)**	121±2	135±3^c^
**Diastolic blood pressure (mm Hg)**	74±2	79±2^a^
**Glycemia (mmol/l)**	5.33±0.11	6.55±0.22^c^
**Insulinemia (pmol/l)**	61.6±9.1	163.7±17.8^c^
**HbA1c (%)**	5.6±0.06	6.17±0.24^a^
**Total cholesterol (mmol/l)**	6±0.34	5.3±0.25
**HDL cholesterol (mmol/l)**	1.92±0.16	1.63±0.13
**LDL cholesterol (mmol/l)**	3.2±0.26	2.64±0.21
**Triglycerides (mmol/l)**	1.3±0.11	2.42±0.33^c^
**Leptin (µg/l)**	15.3±4.8	15.2±3.11
**Adiponectin (mg/l)**	8.1±0.8	5.3±0.34^b^
**Number of MetS components (%)**		
**0**	36	-
**1**	41	-
**2**	23	-
**3**	-	38
**4**	-	52
**5**	-	10
**Treatments (%)**		
**Oral antidiabetic**	0	41
**Insulin**	0	2
**Antihypertensive**	1	70
**Statins**	2	2

Baseline characteristics of MetS patients (*n* = 21) compared to control subjects (*n* = 17). Subjects were fasted before blood collection. All values are expressed in International System (SI) units.

a *P*<0.05;

b *P*<0.01;

c *P*<0.001.

Consistent with our previous findings [12], we found that total circulating levels of MPs and platelet-, endothelial- and red cell-derived MPs were significantly enhanced when compared with HS MPs (Table S1).

### MetS MPs induce *ex vivo* vascular hypo-reactivity in mouse aorta

**Figure 1:**
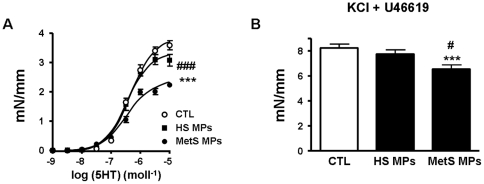
MetS MPs induce vascular hypo-reactivity in mouse aortas. Concentration-response curves to 5-HT (A) and contractile response to the concomitant application of KCl and a single concentration of U46619 (B) in aortic rings from mice treated with either vehicle (CTL, *n* = 7), healthy subject MPs (HS MPs, *n* = 8) or MetS patient MPs (MetS MPs, *n* = 8). ***P<0.001 MetS MPs *vs.* CTL; ## *P*<0.001 MetS MPs *vs.* HS MPs.

5-HT produced a concentration-dependent increase in tension in aortic rings from all groups of mice; however, vascular reactivity to the agonist was markedly decreased in mice treated with MetS MPs compared to those treated with either vehicle or HS MPs (Fig. 1A). Interestingly, the aortas from MetS MP-treated mice, compared to those from vehicle- or HS MP-treated animals, also exhibited an impaired contractile response to the concomitant application of KCl (80 mmoll^−1^) and a single concentration of another vaso-constrictor agonist, thromboxane A_2_ agonist U46619 (100 nmoll^−1^) (Fig. 1B). These data suggest that the MetS MP-induced vascular hypo-reactivity is agonist-independent.

### Involvement of NO in MetS MP-induced vascular hypo-reactivity

**Figure 2:**
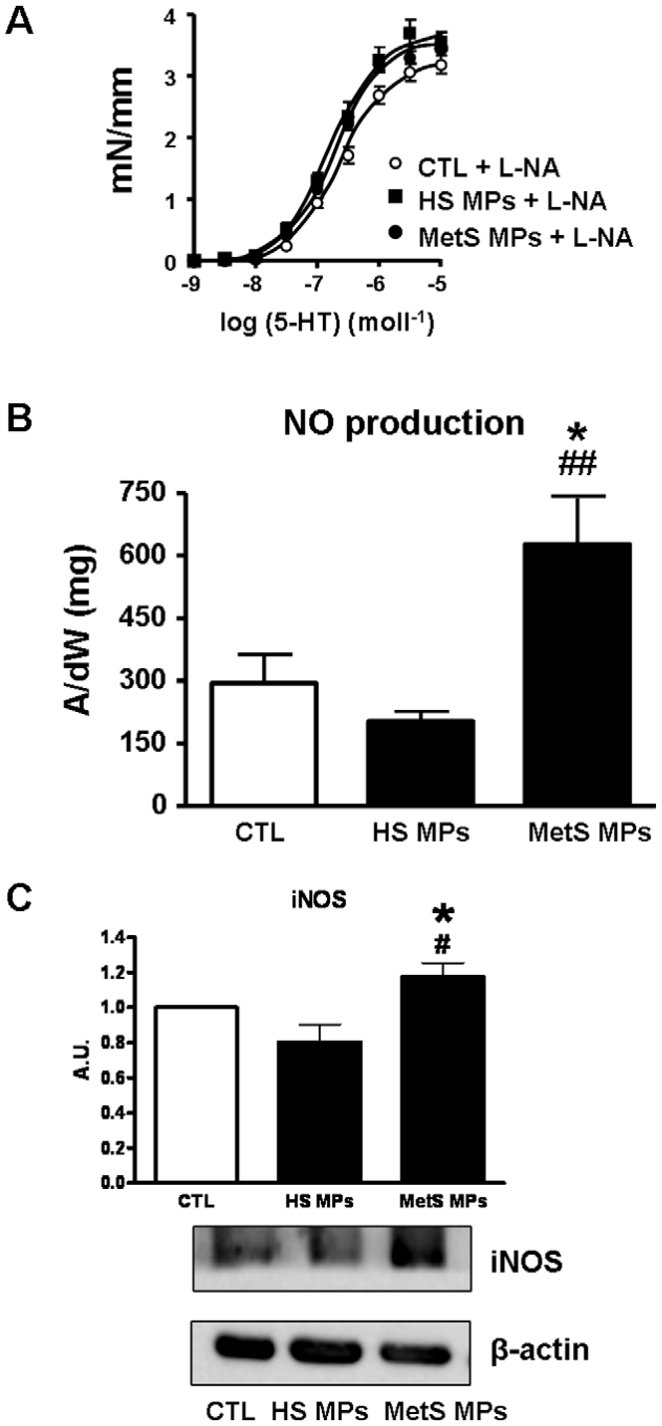
Involvement of NO in MetS MP-induced vascular hypo-reactivity. (A) Concentration-response curves to 5-HT of aortic rings from mice treated with either vehicle (CTL, *n* = 7), HS MPs (*n* = 8), or MetS MPs (*n* = 8) in the presence of NO-synthase inhibitor (L-NA, 100 µmol/l). (B) Quantification of the amplitude of NO-Fe(DETC)_2_ signals in aorta from mice treated with either vehicle, HS MPs or MetS MPs. Values are expressed as units/mg weight of dried (dW) aorta (*n* = 7). (C) Western blot revealing expression of inducible NO-synthase (iNOS) in aorta from mice treated with either vehicle, HS MPs or MetS MPs. Data are expressed as denstometry in arbitrary units (A.U.) as mean ± SEM. * *P*<0.05 *vs.* CTL; # *P*<0.05 MetS MPs *vs.* HS MPs; ## P<0.01 MetS MPs *vs.* HSMPs.

To investigate the role of NO, the effect of the NO-synthase inhibitor, L-NA, was studied in response to 5-HT treatment. Interestingly, we found that inhibition of the NO pathway completely prevented the vascular hypo-reactivity induced by MetS MPs (Fig. 2A), suggesting that NO may be involved in the mechanism of this vascular hypo-reactivity. Direct *in situ* measurements of NO production were performed by EPR spectroscopy using Fe(DETC)_2_ as a spin trap. Aortas from vehicle, HS MP- and MetS MP-treated mice, exhibited an EPR feature of signals derived from NO-Fe(DETC)_2_. The NO-Fe(DETC)_2_ EPR signal was greater in aortas from MetS MPs-treated mice compared to vehicle- and HS MP-treated mice (Fig. 2B). Moreover, MetS MPs markedly increased iNOS expression in mouse aorta compared to vehicle or HS MPs (Fig. 2C) indicating elevated enhanced NO production.

### MetS MPs increase ROS production and increase NADPH oxidase expression

**Figure 3:**
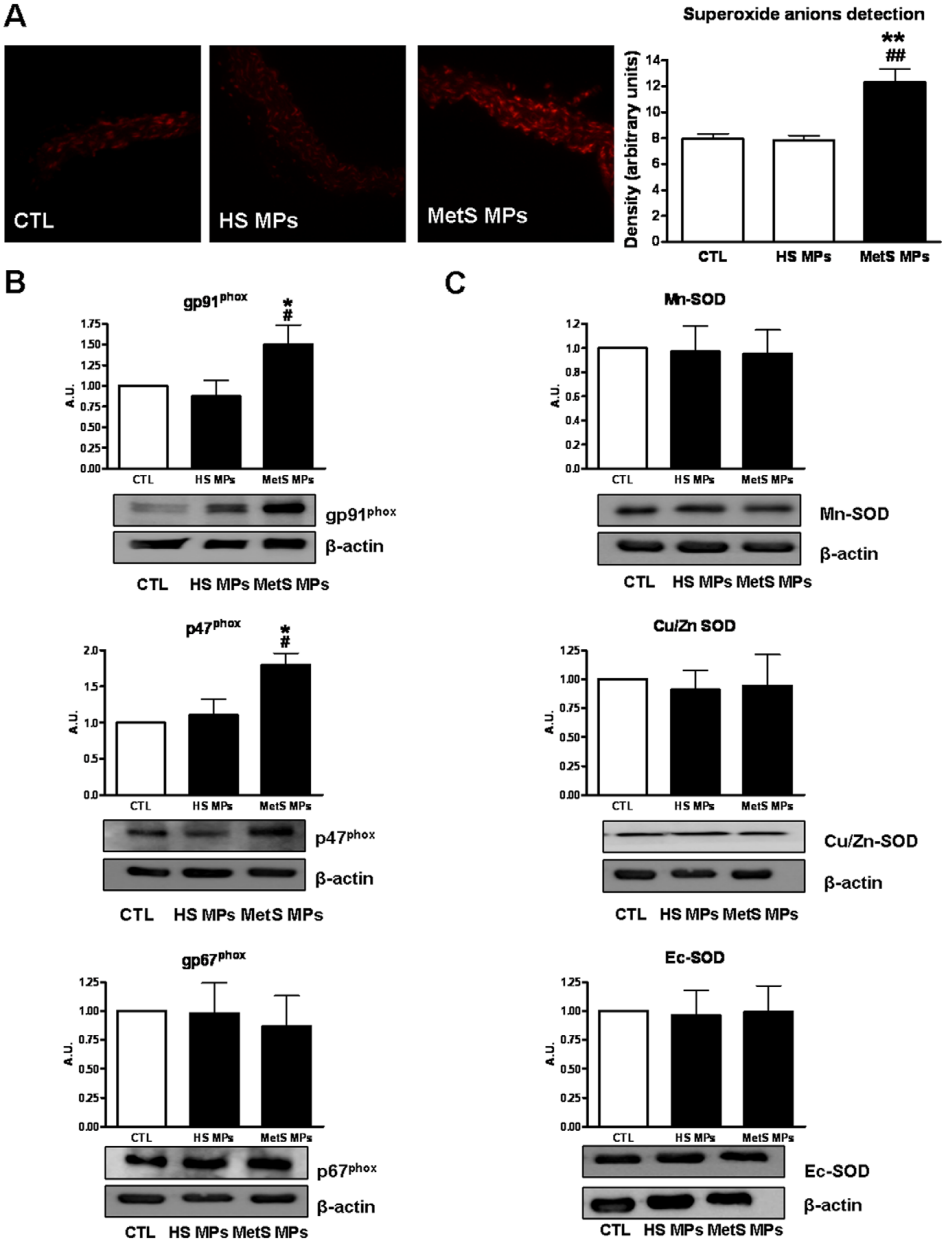
MetS MPs increase ROS production and enhance NADPH oxidase expression. (A) Detection of ROS production in the vascular wall. Vessel sections of aorta from mice treated with either vehicle, HS MPs or MetS MPs were incubated 30 min with DHE and visualized by confocal microscopy. Results are representative pictures of three independent experiments. Bargraphs show the quantification of ROS. Typical examples of ROS staining in each group are shown. (B and C) Western blots revealing expression of NADPH oxidase subunits (gp91^phox^, p47^phox^ and p67^phox^) (B) and expression of SOD isoforms (Mn-SOD, Cu/Zn-SOD and Ec-SOD) (C) in aorta from mice treated with either vehicle, HS MPs or MetS MPs. Data are expressed as denstometry in arbitrary units (A.U.) as mean ± SEM. * *P*<0.05 *vs.* CTL; ** *P*<0.01 *vs.* CTL # *P*<0.05 *vs.* HS MPs; ## P<0.01 *vs.* HSMPs.

Aorta sections from mice treated with MetS MPs displayed an increase in vascular wall (endothelium and in the media layer) ROS production compared to vessels from vehicle or HS MP-treated mice, measured through EtBr fluorescence (Fig. 3A).

We evaluated expression of membrane (gp91^phox^) and cytosolic (p47^phox^ and p67^phox^) subunits of NADPH oxidase, a major source of cellular superoxide anion (O_2_^−^). Interestingly, MetS MPs markedly enhanced the expression of gp91^phox^, as well as p47^phox^ without affecting the level of p67^phox^ in mouse aorta (Fig. 3B), accounting probably for an increase in NADPH oxidase activity, while vehicle or HS MPs produced no effect. To evaluate the capacity of the vessels to reduce O_2_^−^ in the presence of MPs, we examined expression of different isoforms of SOD. MPs from both HS and MetS patients did not significantly affect Mn-SOD, Cu/Zn-SOD or Ec-SOD expression compared to the vehicle control (Fig. 3C).

### Involvement of COX metabolites in MetS MP-induced vascular hypo-reactivity

To investigate the role of COX metabolites in 5-HT-induced vaso-reactivity, the effects of both a non-selective inhibitor of COX (indomethacin) and a selective inhibitor of COX-2 (NS398), were examined.

**Figure 4:**
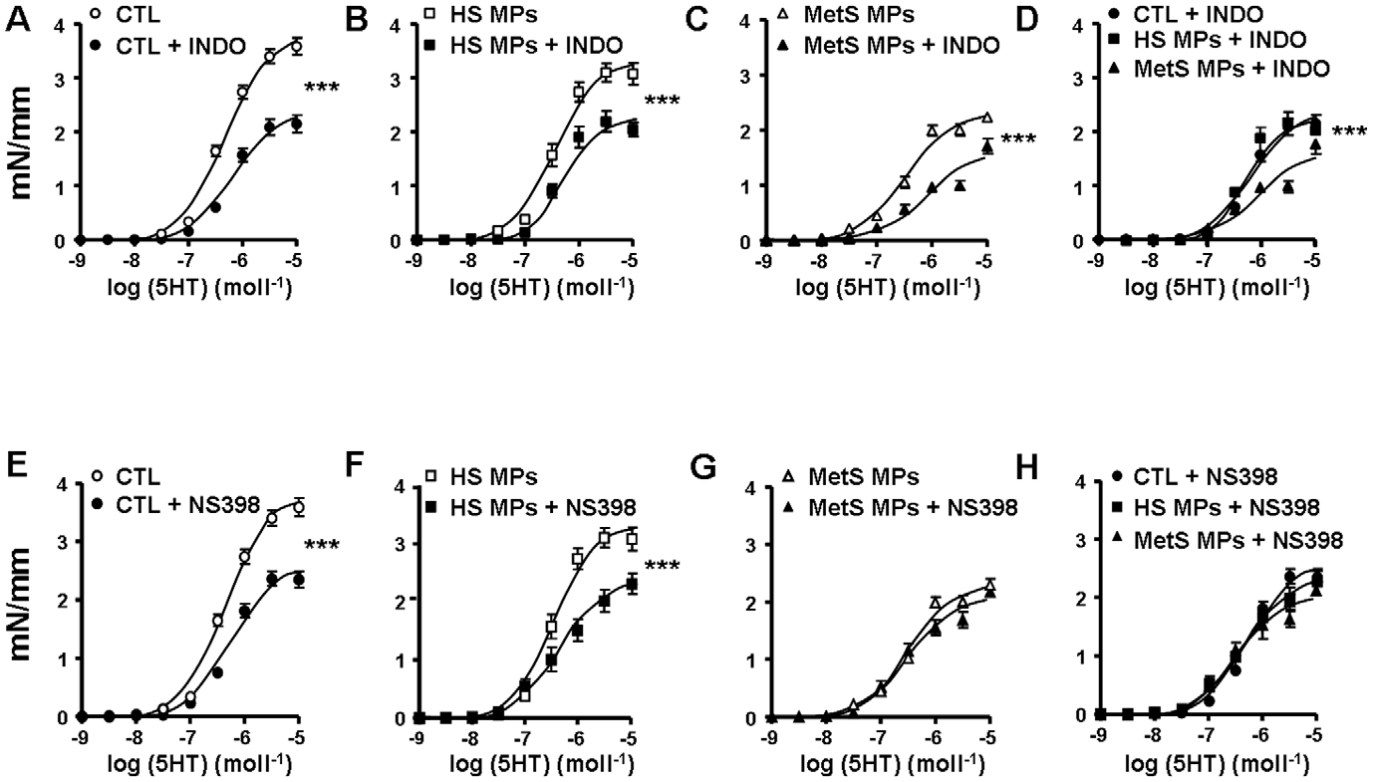
Involvement of COX metabolites in MetS MP-induced vascular hypo-reactivity. (A–D) Concentration-response curves to 5-HT of aortic rings from mice treated with either vehicle (CTL, *n* = 7), HS MPs (*n* = 8) or MetS MPs (*n* = 8) in the absence or presence of COX inhibitor (indomethacin, 10 µmol/l). (E–H) Concentration-response curves to 5-HT of aortic rings from mice treated with either vehicle (CTL, *n* = 7), HS MPs (*n* = 8) or MetS MP (*n* = 8) in the absence or presence of COX-2 inhibitor (NS398, 1 µmol/l). ***P<0.001 *vs.* absence of the inhibitor; ## *P*<0.01 MetS MPs *vs.* CTL.

**Figure 5:**
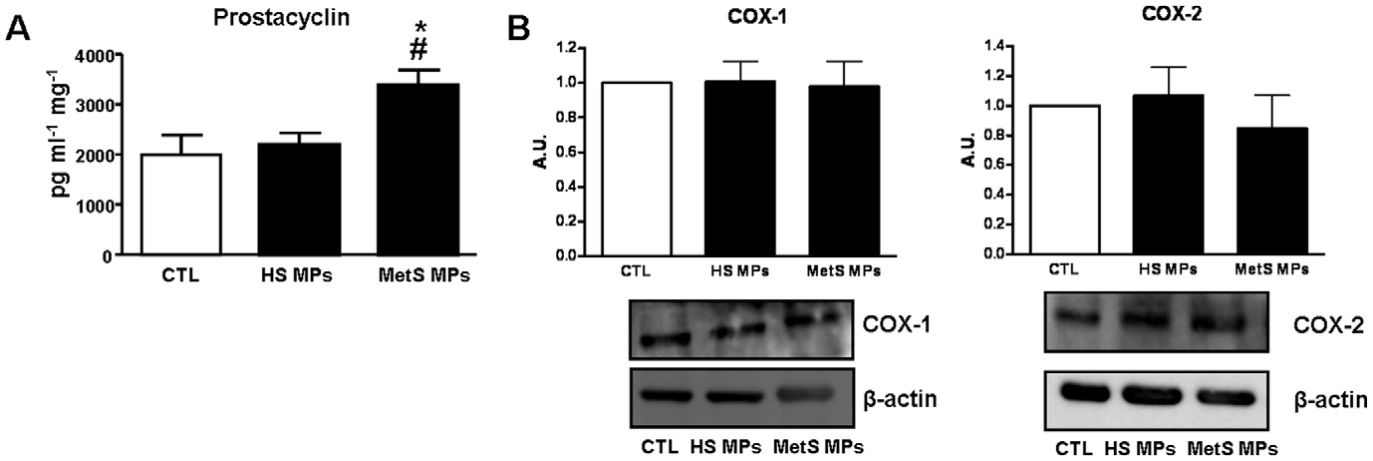
MetS MPs increase prostacyclin release from mouse aorta. (A) Concentration of the COX derivative, prostacyclin in the supernatants of mouse aorta pre-treated with either vehicle (CTL), HS MPs, or MetS MPs (n values?) and stimulated with 5-HT (*n* = 5). (B) Western blots revealing expression of COX-1 and COX-2 in aorta from mice treated with either vehicle (CTL), HS MPs, MetS MPs. Data are expressed as denstometry in arbitrary units (A.U.) as mean ± SEM. *P<0.05 MetS MPs *vs.* CTL; # P<0.05 MetS MPs *vs.* HS MPs.

In the presence of indomethacin, contractile response to 5-HT was reduced in aortas from all groups of mice (Fig. 4A–4C). Thus, vascular hypo-reactivity to 5-HT was still present in aortas from mice treated with MetS MPs compared to HS MPs or vehicle. These results highlight that vasoconstrictor metabolite(s) sensitive to indomethacin participate in 5-HT-induced contraction (Fig. 4D). When COX-2 was specifically inhibited using NS398, the response to 5-HT was impaired in vessels from vehicle and HS MP injected mice (Fig. 4E–4F), suggesting the contribution of COX-2-derived vasoconstrictor metabolites. By contrast, inhibition of COX-2 did not modify the response induced by MetS MPs (Fig. 4G). These results suggest that MetS MPs treatment leads to the release of both vasodilator and vasoconstrictor metabolites and the former blunted the effect of the latter. Interestingly, MetS MPs enhanced the production of prostacyclin, a vasorelaxant metabolite, independently of modification in either COX-1 or COX-2 expressions (Fig. 5B).

### MetS MPs increase MCP-1 expression in mice aortas

**Figure 6:**
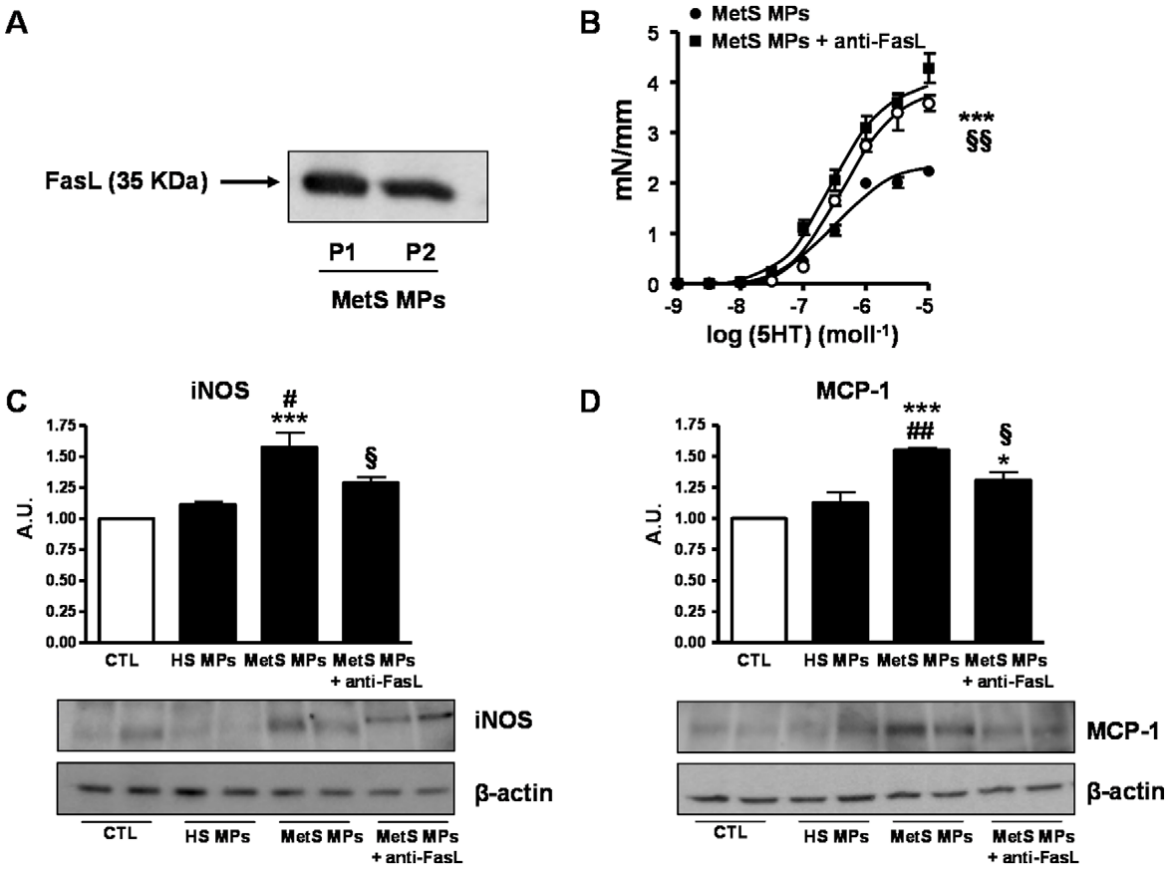
Involvement of Fas/FasL signaling pathway in the MetS MP-induced hypo-reactivity in mouse aorta. (A) Western Blot showing FasL expression in MPs obtained from two different patients (P1 and P2). (B) Concentration-response curves to 5-HT of aortic rings from mice treated with vehicle (CTL, *n* = 7), MetS MPs (*n* = 8), or MetS MPs pre-incubated with FasL antibody (*n* = 6). (C–D) Western Blots showing iNOS (C) and MCP-1 (D) expressions in aorta from mice treated with either vehicle, HS MPs MetS MPs, or MetS MPs pre-incubated with FasL antibody. Data are expressed as denstometry in arbitrary units (A.U.) as mean ± SEM. ***P<0.001 *vs.* CTL; # *P*<0.05 *vs.*HS MPs; *P*<0.001 *vs.*HS MPs; § *P*<0.05 *vs.* MetS MPs + FasL antibody; §§ *P*<0.01 *vs.* MetS MPs + FasL antibody.

In order to evaluate the effect(s) of MPs on inflammatory processes, we analyzed the mRNA expression of pro-inflammatory cytokines. As shown in Table 2, MetS MPs increased the expression of MCP-1, a potent agonist of monocytes, memory T cells, and basophils. However, MPs either from MetS patients or HS had no effect on mRNA expression of TGF-β1, TGF-β2 and TGF-β3 (Table 2). The mRNA expression of the other cytokines (IL-6, IL-8, IL-1α, IL-1β, IL-10 and TNF-α) was not detectable under the experimental conditions used. MCP-1 mRNA increase was reinforced by our observation that aortas from mice treated with MetS MPs exhibited enhanced MCP-1 protein expression compared to those from mice treated either with vehicle or HS MPs (Fig. 6D).

**Table 2:** Quantitative real time RT-PCR analysis.

	mRNA normalized relative quantity (×10^3^)		
	CTL	HS MPs	MetS MPs
**MCP-1**	104±28	51±16	201±17a,b
**TGF-β1**	3226±377	3382±481	2960±331
**TGF-β2**	33±7.5	28.7±6.6	28±7.3
**TGF-β3**	3873±731	3168±485	3600±713

Quantitative real time RT-PCR analysis of normalized relative quantity of IL-6, IL-8, IL-1α, IL-1β, TNF-α, MCP-1, TGF-β1, TGF-β2 and TGF-β3 mRNA expressions in mouse aorta after 24 h of i.v. injection with either vehicle (CTL, *n* = 4), MPs from healthy subjects (HS MPs, *n* = 4) or MPs from MetS patients (MetS MPs, *n* = 4). The mRNA: IL-6, IL-8, IL-1α, IL-1β, IL-10 and TNF-α was not detectable under the experimental conditions used.

a *P*<0.05 *vs*. CTL;

b *P*<0.01 *vs.* HS MPs.

### Involvement of Fas/FasL signaling pathway in the vascular MetS MP-induced hypo-reactivity

Interestingly, we found that MPs from MetS patients expressed FasL (Fig. 6A). When we neutralized FasL harbored by MetS MPs, using specific antibodies, prior to their injection into mice, we completely prevented the hypo-reactivity previously observed in mouse aorta (Fig. 6B). This indicates that Fas/FasL pathway is involved in MP-induced vascular dysfunction. Interestingly, neutralizing FasL also prevented the MetS MP-induced increase of iNOS (Fig. 6C) and MCP-1 (Fig. 6D).

## Discussion

In the present study, we demonstrated that *i.v.* injection of MetS MPs promotes vascular hypo-reactivity in mice. This may be an effect of the release of vasodilatory products from different cellular origins (for example endothelial cells, smooth muscle cells, or fibroblasts). Interestingly, we found that the observed vascular hypo-reactivity was associated with overproduction of vasodilator mediators, such as NO and prostacyclin, as well as increased ROS in the aorta. These effects resulted in an up-regulation of iNOS without changes in COX-1 and COX-2 expression, and enhanced expression of the NADPH-oxidase subunits, gp91^phox^ and p47^phox^. Of particular interest is the finding that Fas/FasL pathway is involved in MetS MP-induced vascular hypo-reactivity. MetS MPs also markedly increased MCP-1 mRNA and protein levels, without changing the mRNA levels of other pro-inflammatory cytokines in the aorta. These data provide valuable information in our understanding of some of the paracrine roles that MPs play as vectors of trans-cellular messengers in promoting vascular dysfunction during MetS.

MPs contribute, at least in part, to the alterations of vascular function in many cardiovascular diseases (for review [7], [9], [32]). In our previous work [12], we showed that MetS patients had increased circulating levels of MPs compared to HS. In particular, we observed an increase in endothelial-, erythrocyte-, and platelet-derived MPs in addition to pro-coagulant (annexin V^+^) MPs in MetS patients. We also reported previously an association between increased circulating MPs and their properties in inducing endothelial dysfunction both *in vitro* and *in vivo*. We showed that the effect of MetS MPs on endothelial cells is driven by non platelet-derived MPs [12]. In the present study, total circulating MPs from HS or MetS patients were injected into mice, in order to mimic the *in vivo* situation where all subpopulations of MPs co-exist. Interestingly, we provide further evidence that MetS MPs are also able to promote vascular hypo-reactivity in response to vaso-constrictor agonists in mice aortas. These findings are in line with our previous studies where MPs generated *in vitro* from apoptotic T lymphocytes or MPs obtained either from diabetic patients [23] or pre-eclamptic women [27], [33] induced vascular hypo-reactivity in mice aortas. In these previous studies, vascular hypo-reactivity was observed even in vessels without functional endothelium. In addition, lymphocyte-derived MPs injected *in vivo* were able to interact directly with smooth muscle as evidenced by an increased CD4 labelling in the media layer of aortas from MP-treated mice compared to control vessels. Furthermore, the same MPs were able to induce iNOS and COX-2 expression in cultured human smooth muscle cells *in vitro* [23], [34]. In another study, *in vivo* injection of mice or *ex vivo* incubation with circulating MPs from pre-eclamptic women reduced contraction to 5-HT in mice aortas [27]. Together, these results suggest that MetS MPs are able to induce the release of vasodilatory factors from smooth muscle cells. However, we cannot exclude that injection of MetS MPs facilitates the *in vivo* interaction between platelets and vessel wall of mice, and the subsequent induction of iNOS and COX-2 in smooth muscle cells that was observed *ex vivo*. Alternatively, MetS MPs can evoke an alteration in the balance between the relaxant and constricting factors in smooth muscle cells.

In regards to vasodilatory factors, vascular hypo-reactivity induced by MetS MPs was accompanied by increased NO production and enhanced expression of iNOS, as well as an increased release of prostacyclin. Interestingly, the inhibition of NOS with L-NA completely prevented MetS MP-induced vascular hypo-reactivity, indicating a crucial role of NO in MetS MP-mediated effects. Previously, we showed that MetS MPs reduced eNOS expression in mouse aortas [12]. Traditionally, the up-regulation of iNOS is thought to compensate for the loss of functional endothelium and eNOS during injury and atherosclerosis [35], although the presence of excess NO and ROS coincidentally may lead to additional tissue damage and dysfunction. Accordingly, Tesse et al. [23] have shown that MPs from T lymphocytes enhanced expression of iNOS and COX-2 with subsequent increased NO and prostacyclin productions, and reduce the vascular contractility to agonists. It has also been shown that MPs from pre-eclamptic women enhance NO production in mouse aortas and that NOS inhibition strongly enhances the response to 5-HT in vessels from mice treated with MPs from pre-eclamptic women [27], [36].

ROS play an important role in the development of cardiovascular diseases, including hypertension, atherosclerosis, stroke, ischemia-reperfusion injury and diabetes [37]. The main enzymatic source of ROS that is important in vascular disease and hypertension is NADPH oxidase [38]. Here, we report that MetS MPs, in addition to increased NO release, led to increases in the expression of membrane (gp91^phox^) and regulatory cytosolic (p47^phox^) subunits participating in the activation of NADPH oxidase activity and O_2_^−^ overproduction. At this stage we are unable to exclude mitochondria as another source of ROS generation.

The aortic contraction in response to 5-HT involved the participation of COX-1 vasoconstrictor metabolites independently of the treatment (vehicle, HS MPs or MetS MPs) indicating that COX-1 metabolites did not affect the capacity of MetS MPs to promote vascular hypo-reactivity. Interestingly, the blockade of COX-2 using a specific inhibitor resulted in a reduced response to 5-HT stimulation in aortas from vehicle-treated and in those from HS MP-treated but not in MetS MP-treated mice. In regards to COX-1 and COX-2 expression, we found no significant differences between aortas taken from mice treated either with vehicle, HS MPs or MetS MPs. These results suggest the existence of a vasoconstrictor metabolite probably from COX-1, which is implicated in the pathway activated by the agonist in mouse aortas independent of MetS MPs treatment inasmuch the same inhibition was observed either in control or in vessels from HS MPs-treated mice. The fact that inhibition of COX-2 caused no effect in vessels from MetS MP-treated mice suggests that this enzyme is not involved in the process. Alternatively, MetS MPs activate COX-2 leading to a release of vasodilator metabolites that are compensated by a release of vasoconstrictor metabolites. The latter hypothesis is supported by the fact that MetS MPs, but not HS MPs, were able to induce an increase in the release of prostacyclin. It is possible that under the conditions studied, prostacyclin may attenuate the vascular damage induced by NO overproduction, and may participate to the adaptive response of vascular cells to MetS MPs.

Evidences from clinical and experimental studies support the hypothesis that inflammation plays an important role in a wide range of cardiovascular diseases and have focused attention on the signals that initiate cellular infiltration of vascular tissues [31], [39]. In the current study, we report that MetS MPs increased vascular expression of MCP-1, which could potentially lead to increased recruitment of leukocytes under conditions associated with vascular inflammation. Previously, we reported that MetS MPs did not affect *in vitro* expression of MCP-1 mRNA in cultured endothelial cells [12]. A possible reason for this discrepancy may be due to additional interactions between MetS MPs and circulating cells following injection into mice.

FasL is a 40-kDa cytotoxic type II trans-membrane protein belonging to the tumour necrosis factor1 family. Unlike Fas, which is constitutively expressed by various cell types, FasL is expressed primarily by cells of the immune system such as activated T cells [18], [40]. Fas and FasL play a crucial role in the induction of apoptosis in various cell types [18], [40]. By deleting auto-reactive lymphocytes, Fas/FasL ensure the development of normal T and B cell repertoires [18], [40], preventing autoimmune disorders. In vascular smooth muscle cells, it has been shown that the activation of the Fas/FasL pathway results in the increased expression of a specific program of inflammatory genes [21], [22]. Functionally, FasL has been shown to be pro-inflammatory, and implicated in pathophysiological processes of various cardiovascular diseases, such as coronary heart disease, arteriosclerosis, and ischemia-reperfusion injury [19]. In human atherosclerosis, FasL is expressed together with markers of apoptosis in inflammatory regions of plaques [41]. FasL-mediated smooth muscle cell apoptosis within the vulnerable plaque may lead to plaque instability and rupture, events well known to cause myocardial infarction and stroke. Given the role of the pro-inflammatory pathway Fas/FasL in the expression of inflammatory genes in smooth muscle cells [21], [22], we hypothesized that the vascular effects of MetS MPs may be mediated by the interaction of FasL, harbored by MetS MPs, and Fas receptor expressed by smooth muscle cells from the vascular wall [41]. In the present study, we find that MetS MPs express FasL and that its neutralization, using a specific antibody, restores the reactivity in vessels from MetS MP-injected mice towards reactivity of aortas either from vehicle- or HS MP-treated mice. Interestingly, neutralization of FasL also normalizes the expression of iNOS and MCP-1 in aortas from MetS MP-treated mice indicating the importance of inflammation and NO in MetS MPs effects. In line with our findings, it has been reported that proapoptotic stimuli, including FasL, or over-expression of Fas-associated death domain protein causes local accumulation of macrophages and triggers transcriptional upregulation of MCP-1 *in vitro* and *in vivo* [21] through a mechanism involving calpains and caspase 8 [22]. Furthermore, Fas/FasL interaction is capable to induce NFκB signalling [40] and may therefore induce iNOS expression [42]. The fact that we found that neutralizing FasL carried by MPs prevented vascular dysfunction supports of the hypothesis of an interaction between FasL from MetS MPs with Fas from the vessel wall of the treated/injected mouse. In support of this, we showed previously that MPs injected *in vivo* are able to interact directly with smooth muscle by increasing CD4 labelling in the media layer of aortas from MP-treated mice compared to vessels from control mice [23].

Interestingly, in the current study we noticed that MetS MPs did not affect the sensitivity to vasoconstrictors as shown by the absence of differences in the pD_2_. Nevertheless, MetS MPs act via other pathways, including but not limited to Fas/FasL, to increase vascular inflammation and enhance release of vasodilator agents (NO, prostacyclins) which participate in decreasing the Emax (hypo-reactivity).

In conclusion, we provide evidence that MetS MPs induce *ex vivo* vascular hypo-reactivity to vasoconstrictor agents in aorta by increasing both oxidative and nitrosative stresses and by increasing the release of COX-2-derived prostacyclin. In addition, we show that MetS MPs increase the expression of MCP-1 mRNA in the vessel wall. The critical role of MPs as a vector of biological messages leading to vascular dysfunction in MetS involving Fas/FasL pathway is also underlined.

These effects of MetS MPs, in addition to their capacity to reduce endothelial vasodilatation, strengthen the notion that MPs cannot only be considered as surrogate markers of endothelial dysfunction or injury, but also as effectors able to amplify pre-existing vascular dysfunction, including vascular hypo-reactivity and inflammation. Thus, MetS MPs may interfere with mechanisms leading to atherosclerotic plaque development and vascular thrombosis during the evolution of MetS.

## Supporting Information

Table S1 **Circulating microparticle levels in patients with metabolic syndrome compared to healthy subjects.** Total MP levels and different populations: platelet- (CD41^+^), endothelial- (CD146^+^), erythrocyte-(CD235^+^) derived and procoagulant (annexin V^+^) microparticles.
(DOC)

## References

[1] Chew GT, Gan SK, Watts GF (2006). Revisiting the metabolic syndrome.. Med J Aust.

[2] Sesti G (2006). Pathophysiology of insulin resistance.. Best Pract Res Clin Endocrinol Metab.

[3] Fulop T, Tessier D, Carpentier A (2006). The metabolic syndrome.. Pathol Biol (Paris).

[4] Magliano DJ, Shaw JE, Zimmet PZ (2006). How to best define the metabolic syndrome.. Ann Med.

[5] Wilson PW, D'Agostino RB, Parise H, Sullivan L, Meigs JB (2005). Metabolic syndrome as a precursor of cardiovascular disease and type 2 diabetes mellitus.. Circulation.

[6] Hugel B, Martinez MC, Kunzelmann C, Freyssinet JM (2005). Membrane microparticles: two sides of the coin.. Physiology (Bethesda).

[7] Martinez MC, Tesse A, Zobairi F, Andriantsitohaina R (2005). Shed membrane microparticles from circulating and vascular cells in regulating vascular function.. Am J Physiol Heart Circ Physiol.

[8] Martinez MC Andriantsitohaina R. Microparticles in angiogenesis: therapeutic potential.. Circ Res.

[9] Martinez MC, Tual-Chalot S, Leonetti D Andriantsitohaina R. Microparticles:targets and tools in cardiovascular disease.. Trends Pharmacol Sci.

[10] Tual-Chalot S, Leonetti D Andriantsitohaina R, Martinez MC. Microvesicles: intercellular vectors of biological messages.. Mol Interv.

[11] Freyssinet JM (2003). Cellular microparticles: what are they bad or good for?. J Thromb Haemost.

[12] Agouni A, Lagrue-Lak-Hal AH, Ducluzeau PH, Mostefai HA, Draunet-Busson C (2008). Endothelial dysfunction caused by circulating microparticles from patients with metabolic syndrome.. Am J Pathol.

[13] Mostefai HA, Meziani F, Mastronardi ML, Agouni A, Heymes C (2008). Circulating microparticles from patients with septic shock exert protective role in vascular function.. Am J Respir Crit Care Med.

[14] Gonzalez-Quintero VH, Smarkusky LP, Jimenez JJ, Mauro LM, Jy W (2004). Elevated plasma endothelial microparticles: preeclampsia versus gestational hypertension.. Am J Obstet Gynecol.

[15] Priou P, Gagnadoux F, Tesse A, Mastronardi ML, Agouni A Endothelial dysfunction and circulating microparticles from patients with obstructive sleep apnea.. Am J Pathol.

[16] Arteaga RB, Chirinos JA, Soriano AO, Jy W, Horstman L (2006). Endothelial microparticles and platelet and leukocyte activation in patients with the metabolic syndrome.. Am J Cardiol.

[17] Chironi G, Simon A, Hugel B, Del Pino M, Gariepy J (2006). Circulating leukocyte-derived microparticles predict subclinical atherosclerosis burden in asymptomatic subjects.. Arterioscler Thromb Vasc Biol.

[18] Nagata S, Golstein P (1995). The Fas death factor.. Science.

[19] Sata M, Suhara T, Walsh K (2000). Vascular endothelial cells and smooth muscle cells differ in expression of Fas and Fas ligand and in sensitivity to Fas ligand-induced cell death: implications for vascular disease and therapy.. Arterioscler Thromb Vasc Biol.

[20] Fukuo K, Hata S, Suhara T, Nakahashi T, Shinto Y (1996). Nitric oxide induces upregulation of Fas and apoptosis in vascular smooth muscle.. Hypertension.

[21] Schaub FJ, Han DK, Liles WC, Adams LD, Coats SA (2000). Fas/FADD-mediated activation of a specific program of inflammatory gene expression in vascular smooth muscle cells.. Nat Med.

[22] Schaub FJ, Liles WC, Ferri N, Sayson K, Seifert RA (2003). Fas and Fas-associated death domain protein regulate monocyte chemoattractant protein-1 expression by human smooth muscle cells through caspase- and calpain-dependent release of interleukin-1alpha.. Circ Res.

[23] Tesse A, Martinez MC, Hugel B, Chalupsky K, Muller CD (2005). Upregulation of proinflammatory proteins through NF-kappaB pathway by shed membrane microparticles results in vascular hyporeactivity.. Arterioscler Thromb Vasc Biol.

[24] Grundy SM, Cleeman JI, Daniels SR, Donato KA, Eckel RH (2005). Diagnosis and management of the metabolic syndrome. An American Heart Association/National Heart, Lung, and Blood Institute Scientific Statement. Executive summary.. Cardiol Rev.

[25] Grundy SM, Cleeman JI, Daniels SR, Donato KA, Eckel RH (2005). Diagnosis and management of the metabolic syndrome: an American Heart Association/National Heart, Lung, and Blood Institute Scientific Statement.. Circulation.

[26] Martin S, Tesse A, Hugel B, Martinez MC, Morel O (2004). Shed membrane particles from T lymphocytes impair endothelial function and regulate endothelial protein expression.. Circulation.

[27] Meziani F, Tesse A, David E, Martinez MC, Wangesteen R (2006). Shed membrane particles from preeclamptic women generate vascular wall inflammation and blunt vascular contractility.. Am J Pathol.

[28] Agouni A, Mostefai HA, Porro C, Carusio N, Favre J (2007). Sonic hedgehog carried by microparticles corrects endothelial injury through nitric oxide release.. Faseb J.

[29] Mostefai HA, Agouni A, Carusio N, Mastronardi ML, Heymes C (2008). Phosphatidylinositol 3-kinase and xanthine oxidase regulate nitric oxide and reactive oxygen species productions by apoptotic lymphocyte microparticles in endothelial cells.. J Immunol.

[30] Vandesompele J, De Preter K, Pattyn F, Poppe B, Van Roy N (2002). Accurate normalization of real-time quantitative RT-PCR data by geometric averaging of multiple internal control genes.. Genome Biol.

[31] Charo IF, Taubman MB (2004). Chemokines in the pathogenesis of vascular disease.. Circ Res.

[32] Mostefai HA, Andriantsitohaina R, Martinez MC (2008). Plasma membrane microparticles in angiogenesis: role in ischemic diseases and in cancer.. Physiol Res.

[33] Tesse A, Meziani F, David E, Carusio N, Kremer H (2007). Microparticles from preeclamptic women induce vascular hyporeactivity in vessels from pregnant mice through an overproduction of NO.. Am J Physiol Heart Circ Physiol.

[34] Tesse A, Al-Massarani G, Wangensteen R, Reitenbach S, Martinez MC (2008). Rosiglitazone, a peroxisome proliferator-activated receptor-gamma agonist, prevents microparticle-induced vascular hyporeactivity through the regulation of proinflammatory proteins.. J Pharmacol Exp Ther.

[35] Hansson GK, Geng YJ, Holm J, Hardhammar P, Wennmalm A (1994). Arterial smooth muscle cells express nitric oxide synthase in response to endothelial injury.. J Exp Med.

[36] Tesse A, Martinez MC, Meziani F, Hugel B, Panaro MA (2006). Origin and biological significance of shed-membrane microparticles.. Endocr Metab Immune Disord Drug Targets.

[37] Pacher P, Beckman JS, Liaudet L (2007). Nitric oxide and peroxynitrite in health and disease.. Physiol Rev.

[38] Paravicini TM, Touyz RM (2008). NADPH oxidases, reactive oxygen species, and hypertension: clinical implications and therapeutic possibilities.. Diabetes Care.

[39] Seizer P, Gawaz M, May AE (2008). Platelet-monocyte interactions–a dangerous liaison linking thrombosis, inflammation and atherosclerosis.. Curr Med Chem.

[40] Ehrenschwender M, Wajant H (2009). The role of FasL and Fas in health and disease.. Adv Exp Med Biol.

[41] Geng YJ, Henderson LE, Levesque EB, Muszynski M, Libby P (1997). Fas is expressed in human atherosclerotic intima and promotes apoptosis of cytokine-primed human vascular smooth muscle cells.. Arterioscler Thromb Vasc Biol.

[42] Aktan F (2004). iNOS-mediated nitric oxide production and its regulation.. Life Sci.

