# Supplementary material for: Microparticles from Patients with Metabolic Syndrome Induce Vascular Hypo-Reactivity via Fas/Fas-Ligand Pathway in Mice
Source: PLoS One. 2011 Nov 15;6(11):e27809. doi: 10.1371/journal.pone.0027809 (PMC3217000; doi:10.1371/journal.pone.0027809)
Supplement: Table S1 — Circulating microparticle levels in patients with metabolic syndrome compared to healthy subjects. Total MP levels and different populations: platelet- (CD41+), endothelial- (CD146+), erythrocyte-(CD235+) derived and procoagulant (annexin V+) microparticles. (DOC) [file pone.0027809.s001.doc]

**Microparticles from patients with metabolic syndrome induce vascular hypo-reactivity via Fas/Fas-Ligand pathway in mice**

Supplemental materials for: Agouni et al.

**Supplemental Table S1. Circulating microparticle levels in patients with metabolic syndrome compared to healthy subjects.** Total MP levels and different populations: platelet- (CD41^+^), endothelial- (CD146^+^), erythrocyte-(CD235^+^) derived and procoagulant (annexin V^+^) microparticles.

|  | Healthy subjects | Metabolic syndrome patients |
| --- | --- | --- |
| Total microparticles/µl plasma | 6614 ± 1189 | 13520 ± 2488^a^ |
| CD41^+^ microparticles/µl plasma | 6276 ± 1191 | 11940 ± 2305^a^ |
| CD146^+^ microparticles/µl plasma | 70.00 ± 8.7 | 117.2 ± 17.7^b^ |
| CD235^+^ microparticles/µl plasma | 161.5 ± 20.8 | 279.6 ± 49.4^a^ |
| Annexin V^+^ microparticles/µl plasma | 1637 ± 453.3 | 4130 ± 1079^a^ |

Results are expressed as events/µl of plasma and given as mean + SEM. ^a^*P* < 0.05, ^b^*P* < 0.01.
